# Dysregulated genes in HIGK-treated *F. nucleatum *and their possible association with HNSCC

**DOI:** 10.22099/mbrc.2024.50171.1982

**Published:** 2025

**Authors:** SB Shanmugam, J Vijayashree Priyadharsini, P Anitha, AS Smiline Girija, A. Paramasivam

**Affiliations:** 1 Clinical Genetics Lab, Centre for Cellular and Molecular Research, Saveetha Dental College & Hospital, Saveetha Institute of Medical and Technical Sciences [SIMATS], Saveetha University, Chennai, India; 2 Department of Microbiology, Centre for Infectious Diseases, Saveetha Dental College & Hospital, Saveetha Institute of Medical and Technical Sciences [SIMATS], Saveetha University, Chennai, India

**Keywords:** Microbes; Cancer; Genotoxicity; Gene expression; Prognosis

## Abstract

The present study aims to identify the differentially expressed genes in HIGK treated with *Fusobacterium nucleatum (Fn) *and their possible role in establishing head and neck squamous cell carcinoma. The study design follows a computational approach wherein multiple databases and tools are used to derive the possible association between *Fn* exposure and the development of HNSCC. The GEOmnibus dataset GSE6927 provided data on the differentially expressed genes in the HIGK treated with *Fn*. The GEO2R analysis revealed 22 differentially expressed genes in HIGK cells treated with *Fn*. The expression profile of these genes was then analyzed in the HNSCC (TCGA, Firehose Legacy) dataset employing the UALCAN database. The present study revealed 5 genes *viz.,* *GSDMD*, *NUP214*, *ZNF426*, *FUT2*, and *SERPINB2 *exhibiting similar expression patterns in *Fn*-treated HIGK and HNSCC datasets. The *GSDMD *and* NUP214 *were found to be upregulated, and the genes* ZNF426*, *FUT2*, and *SERPINB2 *were downregulated. Among the five genes, the *ZNF426 *demonstrated a significant association with the survival of HNSCC patients. The low expression of *ZNF426* presented a poor prognosis compared to the high expression. The study's results identified *ZNF426* as a candidate gene involved in *Fusobacterium nucleatum* infection and HNSCC. Validating this result is necessary to gain insights into the role of the *ZNF426* gene in developing HNSCC. Furthermore, probing the epigenetic factors targeting *ZNF426* can be a potential therapeutic lead.

## INTRODUCTION

Dysbiosis of microbial ecosystems, or 'microbiome,' significantly impacts cancer development and other diseases. Bacteria that are symbiotically linked with the human body, significantly influence the process of tumorigenesis, cancer differentiation, and malignant progression. This influence extends to interacting with established cancer hallmarks, such as inflammation, immune evasion, genome instability, and resistance to anticancer therapies. While extensively studied in the gut, recent attention highlights the role of polymorphic microbes in diverse tissues, mucosal surfaces, and intratumoral environments, including the oral cavity. Carcinogenesis is a process wherein a cell acquires pathogenic or driver mutations that confer growth advantages to the cell, including uncontrolled proliferation and immortality. Exposure to chemical carcinogens induces genotoxicity or DNA damage. Accumulating evidence has demonstrated that pro-carcinogenic microorganisms initiate cancer by producing substances that can be potentially mutagenic. An intricate relationship between cancer and microbial pathogens has been reported for decades. The pathogens such as *Helicobacter pylori, Epstein-Barr virus,* and *Hepatitis B and C *viruses are all classified as Group I human carcinogens by the International Agency for Research On Cancer (IARC) [1]. Exploring mechanisms underlying the initiation and progression of cancer concerning the pathogen-mediated inflammatory processes, activation of oncogenes, and immune evasion can extend our understanding of microbes' role in cancer development. The association of *Helicobacter pylori *[2] and *Epstein-Barr virus* with gastric cancer [3], *Hepatitis B and C viruses *with liver cancer [4], *Fusobacterium nucleatum* with colorectal cancer [5], *Human papillomavirus *with cervical cancer [6], *Kaposi Sarcoma Herpes Virus* with Kaposi Sarcoma [7] and many more have been documented in several studies from across the globe. Reviewing the relationship between the microbial pathogen will provide novel insights and considerations for managing cancer. 

The human microbiota also produces diverse small molecules, including colibactin, a genotoxic molecule from certain strains of *E. coli* [8]*. Fusobacterium nucleatum* is a pro-carcinogenic bacteria that is a normal inhabitant of the oral cavity. Fn is most often associated with inflammation of gingival tissue and other periodontal problems. The organism exhibits its carcinogenic effect by the secretion of adhesins known as Fusobacterial apoptosis protein 2 (Fap2) and Fusobacterium adhesin A (FadA) [9]. Inflammation, once considered to be an antitumoral response, is now projected as an important hallmark of cancer. The microorganisms found near the mucosal surface provide ample opportunity for the resident microbes to evoke a pro-tumorigenic immune response. For example, non-toxigenic *Bacillus fragilis *(NTBF) were found to produce polysaccharide A, which is known to promote regulatory T cell development and alleviate inflammation.

On the other hand, enterotoxigenic *Bacillus fragilis *(ETBF) produced a potent toxin known as fragilysin, a heat-stable metalloprotease activated by cysteine protease fragipain. The activated fragilysin promoted inflammation, resulting in colorectal cancer [10]. Microbial dysbiosis [11] and sustained release of toxic proteins [12] induce cell transformation. The present study has been designed to identify the common differentially expressed genes in Fn-treated HIGK cells and HNSCC patients.

## MATERIALS AND METHODS

**Sample dataset: **The GEOmnibus dataset GSE6927 was used for the study. This series comprised four samples of HIGK (Human Immortalized Gingival Keratinocytes) infected with *Fusobacterium nucleatum *(GSM159481, GSM159482, GSM159483, GSM159484), 4 Sham-infected HIGK cells (GSM159485, GSM159486, GSM159487, GSM159488). Sham-infected HIGK cells served as the control. The HIGK cells infected with *Fn *were taken as the test group (https://www.ncbi.nlm.nih.gov/geo/geo2r/?acc=GSE6927) [13]. Benjamini & Hochberg method was used to derive adjusted p value. An adjusted p-value of less than 0.05 was considered to be significant. The log 2-fold change threshold was set at 1. The pseudogenes and non-coding RNAs were excluded from further analysis.

**Protein-Protein interaction analysis: **The 22 differentially expressed genes (DEGs) were analyzed for their protein-protein interactions using the STRING (Search Tool for the Retrieval of Interacting Genes/Proteins) tool, version 12. The following interaction sources *viz.,* text mining, experiments, databases, co-expression, neighborhood, gene fusion, and co-occurrence were selected for the analysis. The minimum required interaction score was set at 0.400. This bioinformatics resource provides information on direct physical interactions and functional associations between proteins. The nodes in the network represent proteins, while the edges indicate the type of interaction, such as physical, enzymatic, or genetic [14].

**Gene ontology analysis: **The gene ontology analysis was performed using the PANTHER database (v16.0; Protein Analysis Through Evolutionary Relationships). This analysis provided information on the molecular pathways and functions, biological processes, and sub-cellular localization of gene products. A user-defined query of the top 22 genes was fed as a batch to identify the functional classification of genes. Additionally, classification based on pathways was conducted to identify potential pathways associated with the genes [15, 16].

**Gene enrichment analysis: **Understanding large-scale studies involves identifying important biological pathways and protein complexes in complex datasets. Metascape is a user-friendly web portal designed to help experimental biologists analyze and interpret this data type. It integrates diverse biological databases and analytical tools to simplify the process and provide clear results. With features like functional enrichment, interactome analysis, and gene annotation, Metascape makes it easier for researchers to compare data from different experiments [17].

**Gene expression and survival analysis: **The information about the top 22 differentially expressed genes in the Fn-treated HIGK group, as assessed using the GEO2R program, was further investigated in the HNSCC dataset by employing the UALCAN database (http://ualcan.path.uab.edu/cgi-bin/TCGA-survival). The study analyzed 520 samples from patients with HNSCC primary tumors and 44 paired normal samples. The expression profile was measured in transcripts per million (TPM), a standard unit for normalizing RNA-seq data. The significance between different groups was determined by creating box-whisker plots using the TPM values. The study also demonstrated the overall survival of patients with HNSCC using Kaplan-Meier analysis. The high expression and low/medium expression groups were compared to demonstrate the effect of gene expression changes on patients' overall survival [18]. The promoter methylation was also compared between the normal and primary tissue samples.

**Statistical analysis: **The gene expression data from multiple datasets were analyzed using the GEO2R tool. GEO2R uses R packages from Limma to analyse the expression profiling encompassing the microarray data. It presents results as a table and graphic plots [19]. The UALCAN portal analyzed the gene expression profile by comparing the expression levels between groups using the PERL script accompanied by the Comprehensive Perl Archive Network (CPAN) module. The survival plots were generated using “survival” and “survminer” R packages which were further compared by log-rank test. Survival was exclusively used to perform survival analysis, including survival curves, hypothesis tests, and models, whereas, Survminer improved the visualization of Kaplan-Meier and forest plots for clear representation and interpretation of complex survival data [20].

**miRNA expression analysis: **Epigenetic factors such as DNA methylation, modifications in histone proteins, and non-coding RNAs can greatly influence gene expression. Among these factors, microRNAs are thought to be potential drivers of cancer phenotypes and are associated with various types of cancer. By analyzing microRNAs that target the gene with the ZNF426, we may get a clue about the differential expression pattern demonstrated by the gene. The miRBD database is useful for identifying microRNAs targeting specific gene transcripts. Upregulation of microRNA leads to decreased mRNA copies of the target gene, eventually reducing protein expression. Therefore, predicting miRNAs targeting the differentially expressed gene is essential to understanding the role of epigenetic factors in carcinogenesis. The identification of microRNAs targeting *ZNF426* gene transcripts was conducted employing the miRDB database (http://mirdb.org) [21, 22].

## RESULTS

**Figure 1:**
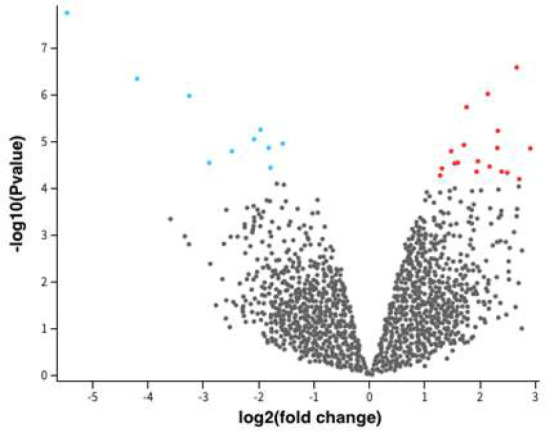
Volcano plot demonstrating differentially expressed genes (DEGs) that are upregulated (red) and downregulated (blue) in *Fusobacterium nucleatum-*treated human immortalized gingival keratinocytes (HIGK). A p-value less than 0.05 is considered significant.

Twenty-two differentially expressed genes (DEGs) were identified, among which 16 genes were found to be downregulated, and 6 genes were upregulated, *viz.*, *FOS*, *GADD45B*, *GSDMD*, *EGR1*,* NUP214*, *NR4A2*. The non-coding RNA, pseudogenes, and repetitive genes were excluded from the DEGs list (Fig. 1). The log2 fold change in the upregulated and downregulated group was found in the range of 1.56 to 5.46 and -1.277 to -2.66. The log2 fold change was higher with *FOS *and lowest in the case of *LIF *genes.

**Figure 2:**
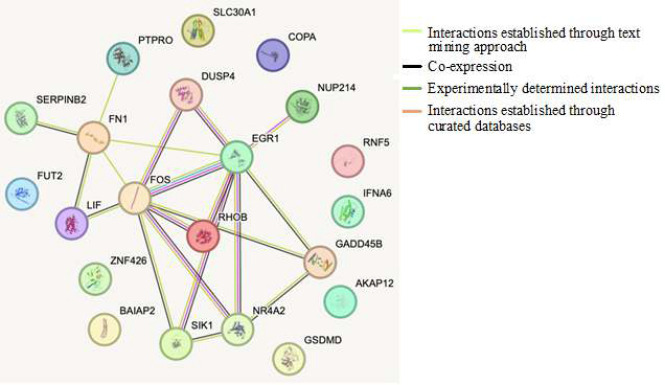
Protein network interactions of the differentially expressed genes (DEGs) as compared using Fn infected-HIGK vs Sham-HIGK cells.

The 22 differentially expressed genes were used as the input query to retrieve the network interactions between the proteins. The proteins RHOB, PTPRO, SERPINB2, FN1, LIF, EGR1, FOS, DUSP4, NUP214, NR4A2, SIK1, and GADD458 were found to exhibit interactions established experimentally from curated databases or through the text mining process. The proteins RHOB, SERPINB2, FN1, LIF, EGR1, FOS, DUSP4, NR4A2, SIK1, and GADD45B were found to be co-expressed. Other network proteins were found to occur independently (Fig. 2). 

**Figure 3:**
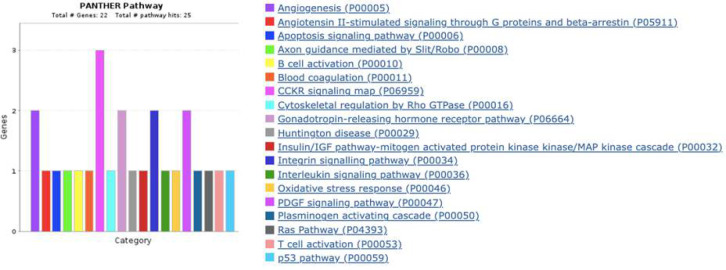
Gene ontology analysis using Panther (http://www.pantherdb.org/) revealed the molecular pathways associated with the differentially expressed genes.

**Figure 4:**
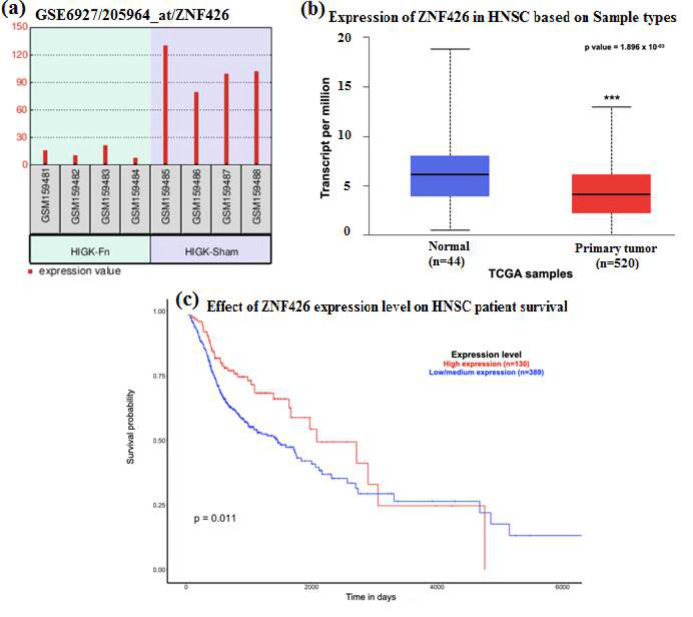
(a) Bar chart demonstrating the expression profile of Fn-infected HIGK vs. Sham-HIGK (b) Box Whisker plot demonstrating the gene expression profile of the *ZNF426* gene in HNSCC datasets. The gene expression between the normal and the HNSCC primary tumor group demonstrated significant downregulation in transcript levels (p=1.896×10^-03^), (c) Kaplan Meier plot demonstrating survival probability of patients presenting with high and low levels of *ZNF426* in HNSCC datasets*.*

Gene ontology analysis for the 22 DEGs revealed several pathways associated with inflammatory processes and carcinogeneses, such as interleukin signaling, angiogenesis, Ras, p53, and integrin signaling pathways (Fig. 3). The highest frequency of genes was found in the CCKR signaling pathway, followed by 2 genes in each pathway leading to angiogenesis, gonadotropin-releasing hormone receptor pathway, apoptosis signaling pathway, and PDGF signaling pathway. All other pathways, as reported by the gene ontology analysis, had at least one gene assigned to it. The Metscape analysis also revealed that the genes associated with conditions of adult fibrosarcoma (Fig. 4). 

*15 genes belonging to unclassified category were removed

The differentially expressed genes were used as a query to identify the gene expression pattern in the HNSCC dataset. Among the 22 genes, the following genes *viz., GSDMD, ZNF426, FUT2, NUP214, *and *SERPINB2* were found to exhibit similar patterns of gene expression in both the HIGK-Fn treated cells and HNSCC dataset. The GSDMD (Gasdermin D; p=0.021966 [HIGK-Fn]; p=1.62×10^-12 ^[HNSCC]) and NUP214 (Nucleoporin 214; p=0.032208 [HIGK-Fn]; p=5.62×10^-13 ^[HNSCC]) were found to be upregulated in both the groups. Whilst, ZNF426 (Zinc finger protein 426; p=0.021966 [HIGK-Fn]; p=1.89×10^-03 ^[HNSCC]), FUT2 (Fucosyltransferase 2; p=0.032208 [HIGK-Fn]; p=1.77×10^-05 ^[HNSCC]), SERPINB2 (Serpin family B member 2; p=0.032208 [HIGK-Fn]; p=2.51×10^-03 ^[HNSCC]) were downregulated in both the groups (Tables 1 and 2). 

The Kaplan-Meier survival analysis demonstrated a significant change in the survival status of the HNSCC patients presenting with decreased expression of Zinc finger protein 426 (p=0.011) (Fig. 4). A statistically significant difference in the ZNF426 methylation profile was observed between the primary tumor and normal samples (p=1.62×10^-12^). There was a significant reduction in the methylation status of primary tumor samples (Median value of 0.043) when compared to normal tissues (Median value=0.046) (Data not shown).

**Figure 5:**
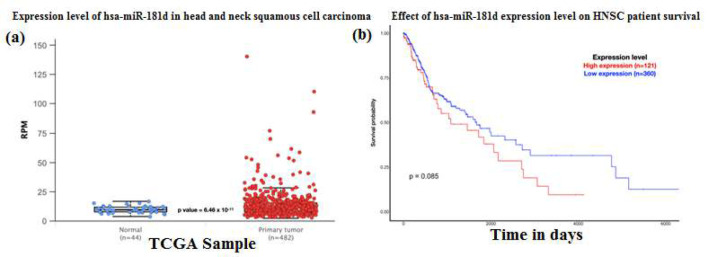
(a) Box Whisker plot demonstrating the gene expression profile of the *hsa-miR-181d* in HNSCC datasets. The gene expression between the normal and the HNSCC primary tumor group demonstrated significant upregulation in transcript levels (p = 6.46 × 10^-11^), (b) Kaplan Meier plot demonstrating survival probability of patients presenting with high and low levels of *ZNF426* in HNSCC datasets*. *The patients exhibiting high expression of *hsa-miR-181d* were found to have a poor prognosis (p=0.085) compared to the low expression group.

The present study identified top 20 microRNAs targeting the ZNF426 gene (Table 3). Among the top 20 microRNAs investigated for their gene expression profile in the HNSCC dataset, *has-miR-181d *was upregulated (p=6.46×10^-11^) in the primary tumor tissue (Fig. 5). This microRNA also exhibited a marginal association with the survival of HNSCC patients (p=0.085). The patients presenting with increased expression of *hsa-miR-181d *showed poor prognosis. Although not statistically significant, the observation of the impact of microRNAs on the expression of the target gene has been elucidated well.

**Figure 6:**
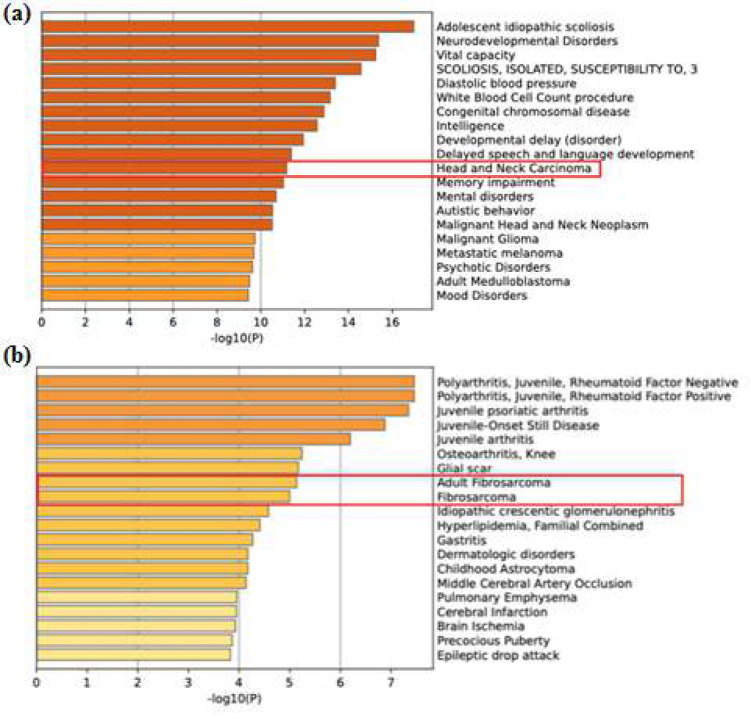
Gene enrichment analysis of potential gene targets of (a) *hsa-miR-181d-5p *and (b) *hsa-miR-181d-3p*

The gene enrichment analysis employing Metascape revealed the enriched pathways associated with the DEGs identified. Cytokine signaling in the immune system showed significant enrichment, indicated by a higher log(10) p-value, and intracellular protein transport showed the least log(10) p-value. Other pathways, such as TGF beta signaling, toll-like receptor cascades, vasculature development, and Rho GTPase cycle, have been established as key pathways involved in carcinogenesis. The GE analysis for the potential targets of *hsa-miR-181d-3p* (39 predicted targets) and* 5p *(1408 predicted targets) revealed enrichment of genes for head and neck cancer and fibrosarcoma respectively. This observation provides evidence of the putative role of *hsa-miR-181d *in HNSCC (Fig. 6).

## DISCUSSION

The present study revealed the differential expression profile of candidate genes in the keratinocytes infected with *Fusobacterium nucleatum *and their concomitant expression in the head and neck cancer group. There were 22 DEGs identified to be associated with inflammatory and tumorigenesis pathways, of which five genes *viz., GSDMD, ZNF426, FUT2, NUP214, *and *SERPINB2 *showed similar expression patterns in the Fn-treated HIGK group and HNSCC datasets. Probing further into how the differential expression status influences the survival of HNSCC patients, we demonstrated that *ZNF426 *was the only gene among the five common DEGs to influence patients' survival status significantly. Most patients presented with low/medium expression (N=389) of ZNF426, which correlated with poor prognosis compared to the high expression group (N=130). The possible reason for this observation can be attributed to the epigenetic components that are known to modify the gene expression process. Given this, the expression of microRNAs targeting the *ZNF426 *gene was investigated. Among 20 microRNAs selected, one was significantly upregulated with a near-positive correlation with the survival of HNSCC patients (p=0.085). Therefore, the present study provides insights into the role of epigenetic components influencing the gene expression process with a special emphasis on microRNAs. 

**Table 1:** List of differentially expressed genes of the GSE6927 dataset (Sham-HIGK vs. FN treated -HIGK)

**Gene symbol**	**Protein encoded**	**adj. P-value**	**logFC**
FOS	Fos proto-oncogene, AP-1 transcription factor subunit	0.000386	5.4641924
LIF	Leukemia inhibitory factor	0.002851	-2.6621763
GADD45B	Growth arrest and DNA damage inducible beta	0.003306	4.1962632
DUSP4	Dual specificity phosphatase 4	0.004614	-2.14
SIK1	Salt inducible kinase 1	0.006715	-1.7573836
BAIAP2	BAI1 associated protein 2	0.016143	-2.3215385
GSDMD	Gasdermin D	0.021966	1.5648093
RHOB	Ras homolog family member B	0.021966	-1.710366
EGR1	Early growth response 1	0.021966	1.8180771
FN1	Fibronectin 1	0.021966	-2.3120466
ZNF426	Zinc finger protein 426	0.021966	-2.908895
PTPRO	Protein tyrosine phosphatase, receptor type O	0.0222	-1.4782003
FUT2	Fucosyltransferase 2	0.032208	-1.9630599
COPA	Coatomer protein complex subunit alpha	0.032208	-1.5997713
NUP214	Nucleoporin 214	0.032208	2.8938432
SERPINB2	Serpin family B member 2	0.032208	-1.5409808
RNF5	Ring finger protein 5	0.03582	-2.1722288
NR4A2	Nuclear receptor subfamily 4 group A member 2	0.035849	1.7882259
AKAP12	A-kinase anchoring protein 12	0.035849	-1.3120649
TESMIN/MLT5	Testis expressed metallothionein like protein	0.038691	-1.9390961
IFNA6	Interferon alpha 6	0.039347	-2.4907742
SLC30A1	Solute carrier family 30 member 1	0.043165	-1.2778307

**Table 2:** Gene expression profile of DEGs in FN treated-HIGK and HNSCC dataset (TCGA)

**Gene symbol**	**Gene expression in PG treated HIGK**	**Gene expression in HNSCC**	**P value**	**Survival **
FOS	Upregulated	Downregulated	9.292 x 10-11	0.44
LIF	Downregulated	Upregulated	1.196 x 10-02	0.056
GADD45B	Upregulated	Downregulated	4.775 x 10-03	0.081
DUSP4	Downregulated	Insignificant	8.262 x 10-01	0.53
SIK1	Downregulated	Insignificant	8.243 x 10-01	0.73
BAIAP2	Downregulated	Insignificant	8.845 x 10-01	0.33
GSDMD	Upregulated	Upregulated	1.624 x 10-12	0.84
RHOB	Downregulated	Upregulated	7.199 x 10-03	0.36
EGR1	Upregulated	Downregulated	8.277 x 10-05	0.81
FN1	Downregulated	Upregulated	1.624 x 10-12	0.051
ZNF426	Downregulated	Downregulated	1.896 x 10-03	0.011
PTPRO	Downregulated	Upregulated	1.096 x 10-09	0.44
FUT2	Downregulated	Downregulated	1.771 x 10-05	0.11
COPA	Downregulated	Upregulated	1.724 x 10-12	0.084
NUP214	Upregulated	Upregulated	5.628 x 10-13	0.27
SERPINB2	Downregulated	Downregulated	2.516 x 10-03	0.72
RNF5	Downregulated	Upregulated	8.808 x 10-10	0.82
NR4A2	Upregulated	Insignificant	1.551 x 10-01	0.12
AKAP12	Downregulated	Insignificant	8.960 x 10-02	0.52
MLT5	Downregulated	Upregulated	1.624 x 10-12	0.29
IFNA6	Downregulated	NA	NA	NA
SLC30A1	Downregulated	Upregulated	7.248 x 10-12	0.51

**Table 3:** The list of top 10 microRNAs targeting the *ZNF426* gene

**Target Rank**	**Target Score**	**Gene Symbol**	**Gene expression profile**	**P-vlaue**	**Survival (p-value)**
1	100	hsa-miR-3925-3p	Data not available	NA	NA
2	100	hsa-miR-188-3p	Upregulated	1.86 x 10-7	0.14
3	100	hsa-miR-4262	Data not available	NA	NA
4	99	hsa-miR-181b-5p	Upregulated	8.36 x 10-10	0.99
5	99	hsa-miR-4302	Data not available	NA	NA
6	98	hsa-miR-181c-5p	Downregulated	2.63 x 10-02	0.22
7	98	hsa-miR-181d-5p	Upregulated	6.46 x 10-11	0.085
8	98	hsa-miR-181a-5p	Upregulated	<10-12	0.59
9	98	hsa-miR-6873-3p	Data not available	NA	NA
10	98	hsa-miR-766-3p	Upregulated	1.62 x 10-12	0.91
11	98	hsa-miR-4768-5p	Upregulated	6.78 x 10-03	0.94
12	98	hsa-miR-6833-3p	Upregulated	9.83x 10-02	0.96
13	97	hsa-miR-1260a	Data not available	NA	NA
14	97	hsa-miR-1260b	Insignificant	4.76 x 10-01	0.65
15	97	hsa-miR-6516-3p	Insignificant	6.13 x 10-01	0.1
16	97	hsa-miR-3156-3p	Data not available	NA	NA
17	96	hsa-miR-1279	Data not available	NA	NA
18	96	hsa-miR-6126	Data not available	NA	NA
19	96	hsa-miR-6845-3p	Data not available	NA	NA
20	95	hsa-miR-199a-5p	Downregulated	3.90 x 10-02	0.98

Numerous research has shown a growing interest in comprehending the effects of inflammation on cancer initiation and progression, focusing mainly on *Helicobacter pylori*-induced inflammation in gastric cancer and *Fusobacterium nucleatum*-triggered inflammation in colorectal cancer. *Fusobacterium nucleatum*, first discovered in the oral cavity, has been linked to oral squamous cell carcinoma development. Studies have shown its potential to promote cancer by activating cell proliferation, promoting cellular invasion, inducing chronic inflammation, and evading the immune system [23]. Inflammation is a key factor in all stages of tumor development. Inflammasomes, part of the innate immune system, regulate immune cells by controlling cytokine and chemokine release, thus impacting anti-tumor immunity. Inflammasomes vary across tumor types and stages, playing distinct roles in progression. Understanding inflammasome function and regulation can lead to innovative cancer therapies, including new drug targets. We identified Gasdermin D as an upregulated gene in bacteria-treated cells and HNSCC [24]. Liu and their team conducted a study to examine the expression and significance of GSDMD-N and NLRP3 in breast cancer. They analyzed 90 breast cancer tissue samples to understand the association between pyroptosis effectors GSDMD-N and NLRP3. The results of the study showed that higher expression levels of these proteins are associated with an improved prognosis and lower risk of death in breast cancer patients. The findings suggest that NLRP3 and GSDMD-N expression levels in breast cancer tissues could serve as an indicator of tumor prognosis [25]. Another study examined the role of three proteins, Nod-like receptor protein 3 (NLRP3), cysteine-aspartic acid protease 1 (Caspase-1), and Gasdermin D (GSDMD), in nasopharyngeal carcinoma (NPC). The study investigated the relationship between these proteins' expression and NPC's recurrence and metastasis. According to the study, the positive expression of NLRP3, Caspase-1, and GSDMD is an independent protective factor against recurrence and metastasis. These proteins can inhibit the proliferation, invasion, and migration of NPC cells by promoting cell pyroptosis [26]. 

Nucleoporin 214 was the other gene that was upregulated in both study groups. Recent studies have identified two nuclear pore proteins, Nup214 and Nup88, as negative regulators of Notch signaling. These proteins control the nuclear export of RBP-J, which is crucial for the proper functioning of Notch signaling. This pathway plays an important role in the organism's development by orchestrating the cells' fate. It is also implicated in the process of carcinogenesis. The loss of Nup88/214 enhanced Notch activity, which shows their role in fine-tuning the cellular signaling process. In addition, the NUP214 fusion protein was found to contribute to tumorigenesis in cases of T-cell acute lymphatic leukemia [27]. A study aimed to investigate the role of the SET-NUP214 fusion gene in measuring residual disease (MRD) after allogeneic hematopoietic stem cell transplantation (allo-HSCT) in patients with acute leukemia. The analysis of 24 cases revealed that SET-NUP214 expression was linked to leukemia relapse after transplantation. Patients who tested positive for SET-NUP214 showed a higher 2-year cumulative incidence of relapse, indicating its potential as an MRD marker for post-transplant prognosis in acute leukemia. ROC curve analysis demonstrated the diagnostic significance of SET-NUP214 in evaluating MRD status after allo-HSCT [28]. Another study conducted by Bhattacharjya and team unravels the inhibition of NUP214 by miR-133b, thereby inducing early mitotic delay leading to chromosomal defects and cell death. All these studies provided substantial evidence of the role of NUP214 as a tumor-promoting gene and that inhibiting its expression can regulate the process of cell death [29].

The blood group antigens, specifically histo-blood group antigens (HBGA), are controlled by the *FUT2* gene, encoding Fucosyltransferase 2. The expression of *FUT2* influences HBGA in the gut, shaping unique phenotypic profiles in populations with distinct evolutionary histories. The polymorphic mutations in the *FUT2* gene have been linked to various diseases, including potential implications for COVID-19 susceptibility [30]. A recent study investigated the rising incidence of early-onset colorectal cancer (EOCRC) across the world and identified potential risk factors during childhood or adolescence, with a focus on long-term antibiotic use (LRAU) and its interaction with genetic factors. The study analyzed data from the UK Biobank and found a correlation between LRAU and a higher risk of EOCRC and adenoma. The researchers also found that individuals with the rs281377 TT genotype had a greater risk of adenoma when stratified by FUT2 genetic polymorphisms. The study highlights the need for further research on how LRAU, genetics, and microbiome-related pathways collectively affect the risk of EOCRC [31]. Although there was no correlation between the differential expression and the survival of HNSCC patients, the association of the *FUT2 *gene with infectious disease and cancer is worth investigating to get more insight into their involvement in establishing cancer phenotypes. 

*SERPINB2 *encodes Serpin family B member 2, infamously known as the plasminogen activator inhibitor 2, which is significantly overexpressed in conditions of stress and senescence. It was found to be upregulated under inflammatory conditions and diseases related to the immune system [32]. The role of SERPINB2 has been implicated in several cancer types, such as bladder, endometrial, colorectal, and ovarian cancers. A study conducted to identify the molecular determinants of chemoresistance in HNSCC employing genome-wide gene expression analysis revealed SERPINB2 as a pivotal candidate contributing to the drug-resistant phenotype. The downregulation of SERPNB2 was found to reduce the overall survival of HNSCC patients who were exposed to cisplatin-based chemotherapy [33]. The present study also demonstrated the downregulation of SERPINB2 in *Fn*-treated and HNSCC with no influence on the survival of patients, which has to be further probed. 

The association of microbial pathogens with cancer development has been reported for decades, with a special emphasis on viruses. The Human Papillomavirus (HPV), specifically HPV-16, has emerged as a significant factor in the progression of this disease. A research study by Sobocińska and team investigated the role of the KRAB-ZNF gene family in epigenetic suppression, focusing on six ZNF genes *viz.,* *ZFP28, ZNF880, ZNF132, ZNF426, ZNF540, *and* ZNF418 *using *in silico *analyses from TCGA and GEO datasets. The results revealed significant downregulation in tumor tissues, with varied expression linked to HPV status and clinical parameters. Interestingly, overexpression of *ZNF540* in HPV-positive patients emerged as a potential prognostic biomarker, correlating with HPV infection and affecting HNSCC outcomes [34]. A study demonstrated seven genes that confer protection against ovarian cancer. The *ZNF426* was among the seven genes whose upregulation was associated with better survival of cancer patients. The present study also showed that the *ZNF426 *high-expression group presented with a better prognosis than the low-expression group. A conclusion can be derived from the present observation that epigenetic factors such as microRNA and methylation patterns could be the reason for the low expression of *ZNF426. *Although a significant change in gene expression was observed between the primary tumor and normal tissues, the observation did not correlate well with the gene expression profile, thereby shifting the focus towards non-coding RNAs.

The *hsa-miR-181d *was identified as a candidate microRNA demonstrating over-expression in the primary tumor tissues of HNSCC patients. Gene enrichment analysis of* hsa-miR-181d t*argets provided strong evidence of their association with HNSCC. A study by Tsai and team investigated the expression of microRNAs in the oral squamous cell carcinoma tissues. They found a 2.5 - 3 fold increase in the expression of this microRNA in OSCC tissues as assessed using TaqMan Human MicroRNA array [35]*. *Therapeutic targeting of these epigenetic marks could aid in maintaining higher levels of *ZNF426 *transcripts, resulting in a better prognosis for HNSCC patients. Appreciating the merits of the present study being a cost-effective method for identifying prognostic and diagnostic biomarkers related to HNSCC, the study also presents with certain limitations, such as validation of results in different populations, especially the Asian population where the incidence of the disease is high, predominantly due to habitual practices or exposure to carcinogenic chemicals as opposed to infections due to viruses which is more prevalent in American or European population. Therefore, a population-based study or analysis is warranted to understand better the tumorigenic pathway mediated by *Fusobacterium nucleatum. *
